# The Role of Problem-Based Learning Approach in Teaching and Learning Physics: A Systematic Literature Review

**DOI:** 10.12688/f1000research.136339.1

**Published:** 2023-08-08

**Authors:** Gumisirizah Nicholus, Charles Magoba Muwonge, Nzabahimana Joseph

**Affiliations:** 1 African Centre of Excellence for Innovative Teaching and Learning Mathematics and Science (ACEITLMS), University of Rwanda College of Education, Kayonza, Eastern Province, Rwanda; 2 Department of Educational Foundations and Psychology, Mbarara University of Science and Technology, Mbarara, Western Region, Uganda; 3 School of Education, University of Rwanda College of Education, Kayonza, Eastern Province, Rwanda

**Keywords:** Problem-based learning, students’ academic achievement, attitude in Physics, systematic review

## Abstract

Problem-based learning (PBL) is a learner-centered method in which complex real-world problems are used to stimulate students thinking and problem-solving abilities during the teaching and learning process. This systematic review aimed to reveal the role of the PBL approach in teaching and learning physics. Relevant articles for the review were sourced from Scopus and Web of Science using keywords such as “problem-based learning” and “PBL in physics” education” as search terms. This search yielded 376 results. Thirty-six articles were included in the analysis after passing a crucial condition of empirically investigating the effect of PBL in teaching and learning physics. Only three of these articles did not show a positive effect; others have shown a positive lead of PBL towards improving academic achievement, attitude to learn physics, problem-solving, critical and creative thinking abilities, cooperative learning enhancement, mental model development, and science process skills attainment. Therefore, the review offers important pointers to various classroom environments and activities that ignite learners’ thinking. Thus, it help policymakers to select and maintain the best methodology that promotes high students’ academic achievement levels.

## Introduction

Our society needs well-trained human resource personnel with skills like problem-solving, critical thinking, collaboration, communication, and manipulation, which can help them cope with new labor market demands ( Supo, 2019). This approach promotes learning involvement in which problems are used to encourage learners to actively engage in the learning process rather than relying on information provided by the teacher ( Karmila *et al*., 2021). This has facilitated the change from conventional teaching methods to a student-centered approach to improving low levels of achievement in a classroom setting. The teacher acts as a facilitator by simply guiding students as they enjoy the classwork in their groups. Problem-based learning (PBL) helps students recognize their current knowledge, realize the gaps in their knowledge and experience, and bridge them by applying new knowledge ( Nursa’ban *et al*., 2019). Students are kept active throughout the learning process. However, following its applications, it works well when the facilitator monitors learners’ progress in their groups and facilitates the acquisition of skills needed by a 21 ^st^-century learner, as emphasized by social constructivist theory ( Vygotsky, 1934). PBL is characterized by complex, real-world situations that do not have one correct answer ( Suwono and Wibowo, 2018). Learners work in teams to confront the problem to identify learning gaps and develop viable solutions, learners gain new information through self-directed learning, the teacher acts as a facilitator, and problems lead to the development of critical thinking and problem-solving abilities ( Ali, 2019). Savery and Duffy (2001) framed PBL instruction in a constructivist theoretical perspective that underlies it and emphasizes the role of learners in constructing their understanding of the world based on their experiences and interactions with their environment.

### Concept of problem-based learning and its implementation

PBL is not more demanding than currently used methods ( Argaw *et al*., 2017) in terms of resources and time and can be implemented with few resources in a school setting and following time allocation for particular topics. However, group work emphasizes interdependency, individual accountability, and the development of social skills that have been found to promote critical thinking and problem-solving skills. Also, it is argued that teachers need to be supported with continuous professional development to be well-equipped with new teaching and learning methods like PBL ( Kanyesigye *et al*., 2022b). The success of implementation lies on the teachers to use PBL. Students depend on their colleagues as they learn. Problem-based learning has been significantly adopted in many educational fields to promote critical thinking and problem-solve in authentic learning situations ( Yew & Goh, 2016). These fields include; Medical schools, Business, and Engineering. The professionals can use the skills from these disciplines to serve our communities ( Yew & Goh, 2016). A recent study by Luy‐Montejo (2019) suggests that the implementation of PBL in a classroom shall be developed in five stages: *Finding a problem;* an investigation begins when a task is prepared by the teacher and is given to the learners to be done. *Organizing ideas on the problem;* learners investigate the problem and generate ideas and knowledge from various sources; the facilitator poses probing questions to learners to stimulate critical thinking and problem-solving abilities. *The group works;* the teacher facilitates the distribution process of learners to groups of 5-10 or 15-20 ( Kanyesigye *et al*., 2022a). This depends on the task’s nature, the class’s size, and available space. During the activity, the group will discuss a problem, the scribe notes down the answers for the group, and the peacemaker maintains peace while other members give contributions to the problem. *Present findings;* learners present solutions to the problem and receive feedback from peers. The teacher consolidates the learning outcomes and allows them to assess their performance. Learners internalize the characteristics of quality work by their peers. *Generalizing;* problems lead to the development of skills. These skills are useful for solving complex, real-world situations that do not have one ‘right answer.’ These are skills that unlock the world and are sought by employers.

PBL is an educational approach that involves presenting students with complex, open-ended problems that are designed to engage them in critical thinking and problem-solving ( Osman *et al*., 2021). Students work in teams to investigate the problem, gather information, analyze data, and develop solutions. However, there are some potential reasons why PBL may work or not work ( Kirschner *et al*., 2006; Savery & Duffy, 2001; Silver-Hmelo, 2004). It engages students in active learning (PBL encourages students to take an active role in their learning, promoting deep learning and retention of information), develops critical thinking skills (PBL promotes critical thinking skills as students must identify problems, analyze information, and develop solutions), encourages collaboration (PBL requires students to work in teams, which helps develop communication and collaboration skills), relevant to real-world problems (PBL involves solving real-world problems, helping students to understand the relevance and applicability of their learning), and increases motivation (PBL can increase motivation and engagement among students as they feel more invested in the learning process).

There are several factors that can limit the effectiveness of PBL. These include the need for significant guidance from teachers and facilitators to be effective, the time-consuming nature of PBL for both teachers and students, the unsuitability of PBL for certain subjects that require a more structured approach or focus on rote learning, the potential challenges when students lack the necessary background knowledge or skills for effective engagement, and the difficulties in assessing student learning due to the open-ended nature of PBL with multiple possible solutions. Thus, PBL can be a practical educational approach that engages students in active learning, develops critical thinking and collaboration skills, and promotes motivation. However, it requires significant guidance, can be time-consuming, and may not be suitable for all subjects.

### Characteristics and effectiveness of PBL in Physics

Problem-based learning is student-centered as the authentic problem engages learners and stimulates their interests ( Ojaleye *et al*., 2018). Problem-based learning promotes learning involvement in which problems are used to encourage learners to actively engage in the learning process rather than relying on the information provided by the teacher. Learners work in teams to confront the problem, identify learning gaps, and develop viable solutions ( Ali, 2019). Learners gain new knowledge information through self-directed learning ( Wilder, 2015). Therefore, the problems help learners develop problem-solving and critical thinking abilities.

PBL is a method that challenges learners to learn by solving physics problems as applied to our daily life experiences. This method enables learners to be self-directed and acquire lifelong learning skills ( Ali, 2019). It produces critical thinkers and problem solvers as learners integrate physics knowledge and skills from the teaching and learning process. PBL motivates learners to find and use appropriate learning resources. Students perceive physics as a difficult subject ( Argaw *et al*., 2017), but research findings have shown that PBL can help students to achieve more in Physics. Several reviews have been done in other fields other than physics education. For instance, the effectiveness of PBL was reviewed in medical studies ( Koh *et al*., 2008; Neville, 2008) and engineering education ( Boelt *et al*., 2022). Our study builds on Coppens *et al*. (2011) study that systematically reviewed PBL in physics and found that PBL can be effective in promoting student learning outcomes, such as conceptual understanding, depending on several factors, including the quality of the problem, the level of student engagement, and the level of scaffolding and support provided by the teacher. Although researchers did a lot to implement this approach, little is known about which variables are learned under PBL or which skills PBL can accelerate in teaching and learning processes. Therefore, the present study focuses on this research question: What role does the PBL approach have in teaching and learning physics? In other words, we wanted to reveal the effectiveness of the PBL approach in teaching and learning physics. Thus, “role” and “effect” of PBL have been used interchangeably in this manuscript.

## Methods

**Figure 1.**
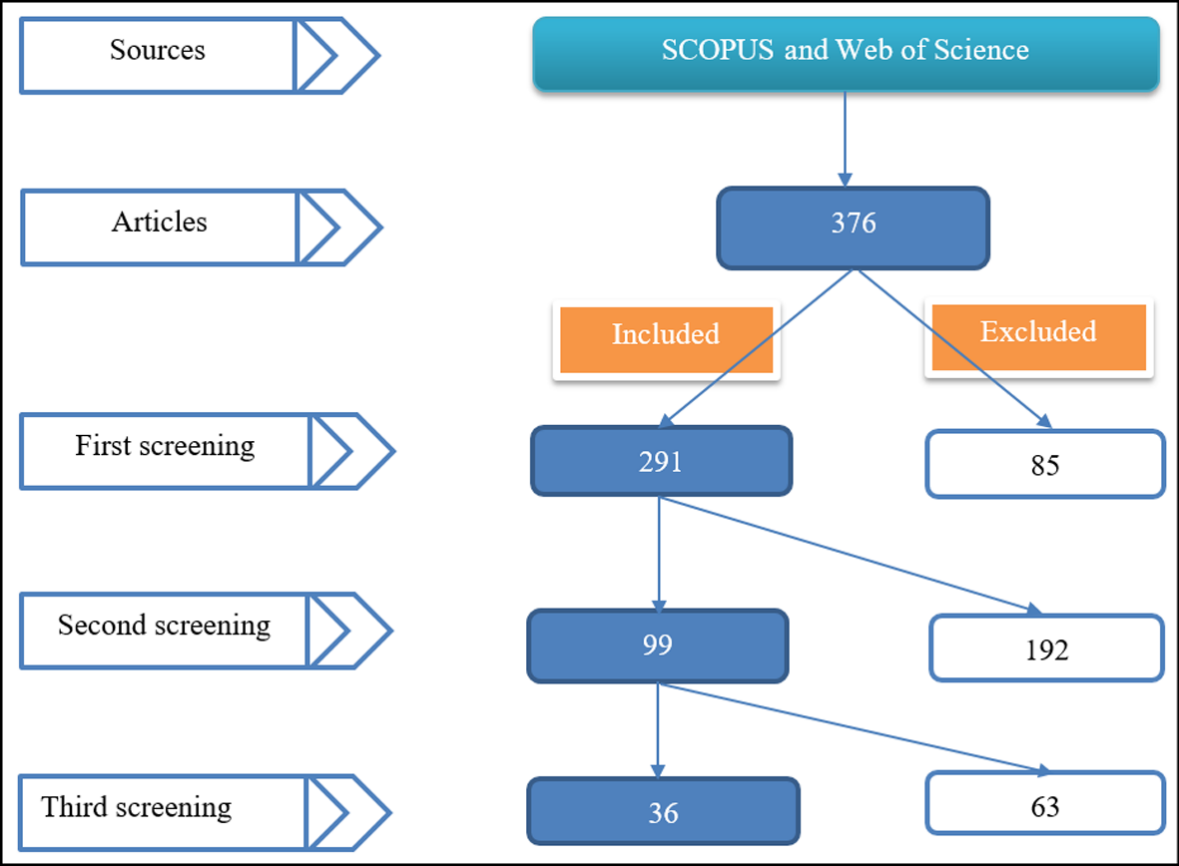
Included and excluded articles.

We used Scopus and Web of Science databases to download articles related to the effect of PBL in learning physics. Two main keywords (problem-based learning and problem-based learning in physics) were used. We used Boolean operators to refine our search (eg., problem-based learning AND problem-based learning physics OR PBL physics OR PBL education NOT medical). “AND” includes both or all words we searched, “OR” included results with either word, but not necessarily both words, and “NOT” excluded articles with that word. We excluded “medical studies” because when we searched for PBL, we found many studies in the medical field. An independently trained research assistant was involved in the search phase to ensure the proper article inclusion. The first author and research assistant independently searched related articles in both databases and shared the output. There was no difference in outputs as they shared common keywords. We initially got 1,914 results from Web of Science Core Collection. We quickly filtered out 295 review articles. We excluded proceeding papers, data papers, and discussions among document types. Among the Web of Science categories, we included articles related to education research and physics education. Thus, we refined by research area (articles related to educational research, physics, optics, astrophysics, biophysics, mechanics, physical geography, and thermodynamics) and also filtered out articles written in other than the English language. This resulted in our final selection and we downloaded 311 articles. Scopus database generated 80 document results. After applying the subject area filter, we were able to download a total of 65 articles [Social Sciences (37), Physics and Astronomy (26), Arts and Humanities (2), Earth and Planetary Sciences (1), and Multidisciplinary (1)]. Thus, 376 articles were used in the first screening (see Figure 1).

The first screening identified 85 duplicates. Thus, 291 were included in the first screening. The second screening identified 99 related to physics after removing articles related to other subjects (102), such as engineering, geography, biology, mathematics, technology, and medicine. The exclusion of 192 articles includes removing general articles (35). These focused on general science, including physics or math and physics. Some articles were also excluded because they did not report the results from primary data (empirically) but were literature reviews.

The third screening comprised of excluding unrelated articles, grey literature, and supplemented approaches. Thus, we first removed 13 unrelated articles, then 32 proceeding papers, and then 19 combined approaches papers. For instance, these “unrelated” articles were related to physics but reported the effectiveness of other approaches, such as inquiry-based learning, project-based learning, mapping-based approach, and problem-solving-based learning. Conference proceedings were excluded in response to appraising the methodological quality of identified articles recommended in systematic reviews. Among studies investigating the role of PBL in teaching physics, 19 were not single; instead, they were supplemented by other approaches. Some were versions of PBL (such as Constructivist PBL, Blended PBL or Hybrid learning model, and Web-based environment), while others were completely different from PBL itself. These are, for instance, Strategic-based learning, 4 Core Areas Model, A Flipped Classroom Approach Using Sigil Software, an e-handout assisted by PhET simulation, a Digital book with 3D animations, Augmented Reality (AR), Video Assistance, Interactive Multimedia in Physics Course, Scientific Approach Based Worksheet, Argumentation Skills, Jukung and Balogo, Android-based physics learning media, Self-Regulated Learning (SRL), E-Books, and Authentic assessment. Thus, 36 articles were found deem to reveal the role of PBL in learning physics and were taken to the final analysis stage.

Data were analyzed in Mendeley 1.19.5 / 2019 software and then exported to MS Excel 2016 to sort variables and produce pivot tables and figures. Each of the 36 studies was analyzed to check what concept or content the study focused on and the skills its PBL improved or did not improve. We then analyzed the participants’ level, the study’s design, and the critical statistical method. We then presented the conclusion of the impact of PBL in teaching and learning physics, checked whether the effect was significant or not, and presented it as positive or no effect. All authors agreed upon a common analysis focus. The first author analyzed the data, while the coauthors validated each step involved. All authors involved in the analysis mostly in identifying the role of PBL in learning physics. Since some forms of effect or role of PBL were in a variety of forms, we coded them in a more understandable and composed way. For instance, students’ achievement, conceptual understanding, and performance were coded as academic achievement. Attitude toward learning physics is code given to motivation to learn physics, appreciation, expectation, beliefs, perception, influence, and like terms. Critical thinking, problem-solving, creative thinking, mental models, cooperative learning, and science process skills were coded as they are.

## Results

Among 36 articles, 16 did not focus on a specific topic or branch of physics; they just mentioned focusing on “physics.” Thus, 20 mentioned a study focus, such as circuit electricity, modern physics, and thermal physics. Many studies were done in mechanics (such as momentum and impulse, material elasticity, and energy), electromagnetism (such as magnetism, circuit electricity, and electromagnetic field), and thermodynamics (such as temperature and heat). Among 36 studies, 23 were investigated university level; twelve were investigated in high schools and one in elementary school. Thirty-three studies used students, while three used teachers as participants.

We identified 13 research designs in reviewed studies, most of which were experimental-related (see Table 1). Specifically, 17 studies were quasi-experimental research designs. Thus, these studies used non-randomized participants in their treatment groups. Two studies used quasi-experimental and observation designs, one used true experimental design (randomly assignment of participants), and three did not mention the type of experiment they used. Seven studies used a survey design, including one exploratory design and one observation checklist (with a rating scale, self, and peer assessment). Other designs (such as action research, correlation, ethnographic, factorial, phenomenography, and Solomon's four-group designs) were used in a single study.

**Table 1.** Research designs among reviewed articles.

	Row labels	Count of research design
1	Action research	1
2	Correlation	1
3	Ethnographic study	1
4	Experimental	3
5	Exploratory	1
6	Factorial	1
7	Observation	1
8	Phenomenographic study	1
9	Quasi-experimental	17
10	Quasi-experimental and observation	2
11	Solomon Four-group	1
12	Survey	5
13	True Experimental	1
	**Grand Total**	**36**

To this end, various analyses were used in respective designs. Descriptive analyses such as percentages and frequencies were used in most of the survey, observation, and exploratory designs. In contrast, inferential statistics (mostly independent-samples t-Test, analysis of variance (ANOVA), analysis of covariance (ANCOVA), effect sizes, and learning gains) were primarily used in experimental studies (see Table 2).

**Table 2.** Data analysis used among reviewed articles.

	Row labels	Count of analysis
1	ANCOVA	6
2	ANOVA	5
3	Descriptive statistics	10
4	Effect size	2
5	Independent-Samples t-Test	4
6	Learning gain	5
7	Linear regression analyses	1
8	Mann–Whitney U test and the Wilcoxon signed-rank test	2
9	Thematic	1
	**Grand Total**	**36**

**Figure 2.**
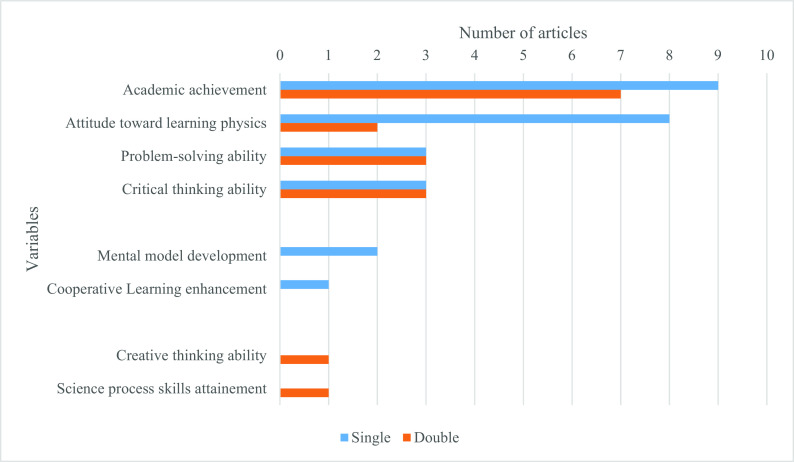
Role PBL instruction.

The role of PBL in learning physics varies from improving student academic achievement and motivation (attitude toward) to learn physics to various skills such as critical thinking and problem-solving ability (see Figure 2). “Single” means that only one of the variables on the vertical axis was integrated into a single study. In contrast, “Double” means that such a variable was investigated with another variable in one study. For instance, nine studies investigated academic achievement alone, while this achievement variable was investigated with attitude toward learning physics in four studies, problem-solving ability, critical thinking ability, and science process skills in one study (each). Generally, attitude toward learning physics with PBL was investigated in ten studies (eight studies alone and two studies with academic achievement and problem-solving ability), the capacity of PBL to develop problem-solving ability in six studies (three studies studied it alone while other three studies studied it in the combination of either academic achievement, attitude toward learning physics or critical thinking ability) and critical thinking ability in six studies (three studies studied it alone while other three studies studied it in a combination of either academic achievement, problem-solving ability or creative thinking ability).

Finally, most of the 36 studies proved a positive effect, except four studies that did not prove a positive effect of PBL in learning physics. Two of these three articles investigated students’ attitudes toward learning physics (specifically, students’ expectations and beliefs about Physics and Physics learning), one for academic achievement (specifically, conceptual understanding), and one for problem-solving skills.

## Discussion

### PBL improves academic achievement

Studying a subject like physics always targets outcomes such as conceptual understanding and academic achievement. However, all these outcomes are correlated because if you understand a particular concept, you will eventually achieve it or get a good score when tested. In this study, several researchers have tested the effect of PBL on students’ academic achievement, and many of these researchers found a positive effect ( Celik *et al*., 2011; Ince, 2012; Kanyesigye *et al*., 2022a; Kartal Taşoğlu & Bakaç, 2014; Pease & Kuhn, 2011; Polanco *et al*., 2004; Savall-Alemany *et al*., 2019; Shishigu *et al*., 2018; Yeo *et al*., 2012). For instance, difficulties in understanding mechanical waves ( Kanyesigye *et al*., 2022a) were remediated by PBL instruction. Based on the Shishigu *et al*. (2018) findings, it was suggested to apply PBL at the college and tertiary level. Because this strategy was helpful for students to scrutinize the connection between theory and practice and eradicate rote memorization, as well as to understand concepts and principles central to physics.

PBL groups was more successful in preventing the determined misconceptions ( Ince, 2012) and more effective than the traditional teaching methods in improving students’ conceptual understanding of magnetism topics ( Kartal Taşoğlu & Bakaç, 2014). Likewise, while PBL students’ improvements in scores were significantly more prominent than control students’ improvements on the Mechanics Baseline Test ( Polanco *et al*., 2004), and there was a significant difference between the two groups in terms of students’ total mean scores in favor of a PBL group, and PBL was found effective on students’ physics achievement ( Celik *et al*., 2011). In their ethnographic study, Yeo *et al*. (2012) investigated the learning journey in PBL in a Physics classroom. They described what happened when a high school physics teacher adopted PBL in his classroom and found that the challenges he faced arose from disparities between the motives driving everyday practices and schooling, which they attribute to differences between academies and the lived realities of practitioners. This is why Pease and Kuhn (2011) found that the effective component of the PBL focuses on engagement with a problem rather than the social component typically associated with the method. Achievement of learning was found to be connected to students’ motivation to learn physics ( Selçuk, 2010), problem-solving ability ( Becerra-Labra *et al*., 2012), and critical thinking ability ( Mundilarto & Ismoyo, 2017).

### PBL increases attitude toward physics and physics learning

In this study, such outcomes from the attitude toward learning physics were effective ( Bergin *et al*., 2018; Kampen *et al*., 2004; Kanyesigye *et al*., 2022b; Maryuningsih *et al*., 2019; Sahin, 2009; Selçuk & Çalişkan, 2010). For instance, results from the study that investigated the effect of PBL on students’ attitudes indicated that the experimental group was more satisfied than the control group ( Selçuk & Çalişkan, 2010). Analysis of student responses indicates that students in the PBL group engaged more in higher-order problem-solving skills and demonstrated a deeper understanding of the learning process than students in the more traditionally hands-on group ( Bergin *et al*., 2018). Teachers appreciated using PBL in the classroom, and the statistical findings indicated a high statistical significance ( Kanyesigye *et al*., 2022b) compared to other teachers that did not receive PBL training. However, the findings from Sahin (2009) suggested further study to investigate predictors and correlates of students’ physics learning using qualitative measures to support and more clearly interpret the numerical finding.

Students’ academic achievement is connected to how students appreciate learning methods such as PBL. In this regard, perception, beliefs, and attitude show beneficiaries’ appreciation of a specific input. It is reasonable to believe that if students excel in a particular subject, they are more likely to develop a positive attitude towards it or experience an increase in their liking for that subject. Such attitudes depend on input, such as the teaching method used, like PBL, and vice versa. When students possess a positive attitude on a specific subject, they will likely be able to perform well ( Heng & Mansor, 2010; Sahin, 2010b). Then, if a new or modernized method improves students’ academic achievement, it will be favored over a traditional method. For instance, group factor ANOVA and one-way ANOVA showed that information literacy treatment affected academic self-efficacy and learning performance ( Heng & Mansor, 2010). The results showed a causal relationship between information literacy training and the improvement of university students’ academic self-efficacy and learning performance in a PBL environment. The authors confirmed that information literacy training could help increase college students’ academic self-efficacy and learning performance, which is essential in the PBL learning process.

### PBL develops critical thinking and problem-solving abilities

Critical thinking and problem-solving are potential skills that students need to possess in this 21 ^st^ century ( Osman *et al*., 2021). We can hypothesize that if a student likes a subject, understands it, and performs on it well, he will eventually possess such potential skills. These two skills were elaborated in this study in many physics studies, the effectiveness of PBL in developing critical thinking ( Jatmiko *et al*., 2018; Parno *et al*., 2019; Wartono *et al*., 2018) and problem-solving ( Jandric *et al*., 2011; Pawlak *et al*., 2020; Yuberti *et al*., 2019). For instance, the results of the effect sizes analysis on the influence of problem-based learning showed a great effect on the critical thinking ability for students in optical instrument topics ( Parno *et al*., 2019). Research on approaching problem-solving skills of momentum and impulse phenomena using problem-based learning showed that the context and problem-based learning (C-PBL) model affected the physics problem-solving skills ( Yuberti *et al*., 2019). A study by Osman *et al*. (2021) showed a relationship between problem-solving ability and critical thinking ability. Science teachers’ experiences study when implementing problem-based learning in rural schools ( Osman *et al*., 2021) indicated that teachers changed their teaching as learners made predictions, formulated hypotheses, and were involved in thought-provoking activities. Problem-solving and critical thinking abilities are critical skills in physics education. Physics is a subject that requires students to apply logical reasoning, mathematical skills, and critical thinking to solve complex problems. The ability to think critically is essential in physics education as it helps students to analyze and evaluate information, identify patterns, and develop a deeper understanding of the subject. Eventually, problem-solving and critical thinking abilities are interconnected. Effective problem-solving requires students to think critically, analyze information, and evaluate potential solutions. At the same time, critical thinking skills are essential for students to develop effective problem-solving strategies and select the best solution.

### PBL develops mental models, science process skills, cooperative learning, and creative thinking

Finally, developing problem-solving and critical thinking abilities can be bettered by attaining science process skills and mental models. The skills required to engage in systematic scientific inquiry are commonly referred to as the scientific process. Enyeneokpon (2012) found a relationship between academic achievement and science process skills. For instance, students exposed to a problem-based learning strategy obtained higher science process skills scores (73.67) than those exposed to the conventional lecture method (26.73). Critical thinking ability and creative thinking ability ( Yanti *et al*., 2022) are both important cognitive skills that are interrelated and complement each other. Critical thinking involves evaluating, analyzing, and synthesizing information to arrive at a logical conclusion or solution. In contrast, creative thinking involves generating novel and original ideas, perspectives, and solutions. A mental model is a cognitive framework or mental representation that individuals use to understand, interpret, and make sense of the world around them. Mental models are based on an individual’s experiences, knowledge, beliefs, and assumptions, and they help shape how individuals perceive and interact with their environment. The fact that PBL showed a positive effect in developing mental models ( Batlolona *et al*., 2020; Batlolona & Souisa, 2020) could be a solution for physics teachers across all levels of education. Overall, PBL was effectively implemented when students learned in cooperative learning groups ( Saka & Kumaş, 2009). This shows the potential of PBL in regard to constructivism learning theory. Constructivism posits that individuals actively construct their own understanding and knowledge through their experiences and interactions with the environment. In PBL, students are presented with a real-world problem or scenario that requires them to apply their knowledge and skills to develop a solution. The problem serves as the starting point for learning, and students are expected to actively engage in the learning process by seeking out information, working collaboratively, and reflecting on their experiences.

## Conclusion and study implication

The studies above suggest that PBL enhances knowledge retention and academic achievement. In addition, there is also a better understanding of physics topics, and students develop critical thinking, problem-solving, and many other skills. Regardless of the specific teaching method used in continuing education, optimizing excitement, maximizing self-efficacy, and minimizing anxiety will help create high levels of student understanding and competence. This is why the theory of constructivism supports the PBL approach. For example, the optimal learning environment for PBL subjects includes teaching that supports reflection and collaboration, sufficient time for independent study, and formative and summative assessments that are tailored to students’ learning problems.

Studies that did not show a positive effect of PBL in learning physics might have been caused by the implementation or study design. A comparison of problem-based learning and traditional lecture students’ expectations and course grades in an introductory physics classroom ( Sahin & Yorek, 2009) and exploring university students’ expectations and beliefs about physics and physics learning in a problem-based learning context ( Şahin, 2009) did not show the effectiveness of PBL on attitude toward learning physics. Both studies probably caused this were in the same project from the same authors. Another study combined attitude and conceptual understanding (students’ epistemological beliefs and conceptual understanding ( Sahin, 2010a), one combined attitude and problem-solving ability (students’ motivation to learn and capacity to build problem-solving skills ( Argaw *et al*., 2017)), and another investigated academic achievement (increasing learning outcomes ( Herliana *et al*., 2020)). Some researchers, such as Hung (2011), have suggested the reasons for some negative outputs. They argued that the concerted efforts of PBL could mitigate questions regarding research methods. However, issues related to PBL implementation have broader implications than simple explanations of unresolved disputes. These things are directly related to the performance of students. Some of the issues are administrative, which is outside of teaching activities. However, some are instructive and can be improved. The recipient is possible to correct unwanted student behavior arising from the PBL process, students’ fundamental way of thinking about teaching methods, and their study habits.

Other variables or skills not covered in the physics body of knowledge are reasoning abilities, metacognitive skills, lifelong learning skills, development of metacognition, self-efficacy, environmental literacy, higher-order thinking skills, management skills, and self-regulated learning. These are covered in other subjects apart from physics. Therefore, future studies should prioritize investigating these aspects. Researchers in physics education are encouraged to explore the effects of Problem-Based Learning (PBL) on additional variables and skills beyond those examined in this study.

## Data Availability

Figshare: Analysis table of findings, https://doi.org/10.6084/m9.figshare.23573958.v1 ( Gumisirizah, 2023). This project contains the following underlying data:
• Analysis table of finding.docx (Studies, Concept, Participants, level of Participants, Research design, Analysis, Role of PBL, Effect) Repository: PRISMA checklist and flow chart for ‘The role of problem-based learning approach in teaching and learning physics: a systematic literature review.’ https://doi.org/10.6084/m9.figshare.23573958.v1 ( Gumisirizah, 2023). Data are available under the terms of the Creative Commons Attribution 4.0 International license (CC-BY 4.0).
