# Supplementary material for: The Role of Problem-Based Learning Approach in Teaching and Learning Physics: A Systematic Literature Review
Source: F1000Res. 2023 Nov 14;12:951. Originally published 2023 Aug 8. [Version 2] doi: 10.12688/f1000research.136339.2 (PMC10646338; doi:10.12688/f1000research.136339.2)
Supplement: Peer review history

# Reviewer report: Reviewer response for version 2

**DOI:** 10.5256/f1000research.158794.r222995

**Published:** 2024-02-22

**Authors:** Elnetthra Folly Eldy

**Affiliations:** 1 Universiti Malaysia Sabah, Kota Kinabalu, Sabah, Malaysia

I am happy to inform you that this paper has been revised and all the comments from the previous review (of mine) have been addressed with utmost diligence. As a result, I am confident that this paper is now ready for publication and will make a significant contribution to the field. Therefore, I recommend that this paper be indexed without any further delay.

Are the rationale for, and objectives of, the Systematic Review clearly stated?

Yes

Is the statistical analysis and its interpretation appropriate?

Yes

Are sufficient details of the methods and analysis provided to allow replication by others?

Partly

Are the conclusions drawn adequately supported by the results presented in the review?

Yes

Reviewer Expertise:

Educational Technology in Physics

I confirm that I have read this submission and believe that I have an appropriate level of expertise to confirm that it is of an acceptable scientific standard.

# Reviewer report: Reviewer response for version 2

**DOI:** 10.5256/f1000research.158794.r222994

**Published:** 2023-11-14

**Authors:** Iqbal Ainur Rizki

**Affiliations:** 1 Universitas Negeri Surabaya, Surabaya, East Java, Indonesia

Thank you for revising this article. I think this article is suitable for indexing.

Are the rationale for, and objectives of, the Systematic Review clearly stated?

Yes

Is the statistical analysis and its interpretation appropriate?

Yes

Are sufficient details of the methods and analysis provided to allow replication by others?

Yes

Are the conclusions drawn adequately supported by the results presented in the review?

Yes

Reviewer Expertise:

Innovative Physics Learning, Technology-assisted Learning, Physics Education

I confirm that I have read this submission and believe that I have an appropriate level of expertise to confirm that it is of an acceptable scientific standard.

# Reviewer report: Reviewer response for version 1

**DOI:** 10.5256/f1000research.149476.r215454

**Published:** 2023-11-03

**Authors:** Iqbal Ainur Rizki

**Affiliations:** 1 Universitas Negeri Surabaya, Surabaya, East Java, Indonesia

The article is scientifically well-structured. It demonstrates consistency in its research questions, methodology, results, discussion, and conclusions. The article also exhibits good originality, with no prior research similar to it. However, in the "Concept of PBL and its implementation" section, particularly concerning the implementation stages, ranging from problem identification to generalization, the inclusion of graphical illustrations would greatly enhance reader comprehension.

Are the rationale for, and objectives of, the Systematic Review clearly stated?

Yes

Is the statistical analysis and its interpretation appropriate?

Yes

Are sufficient details of the methods and analysis provided to allow replication by others?

Yes

Are the conclusions drawn adequately supported by the results presented in the review?

Yes

Reviewer Expertise:

Innovative Physics Learning, Technology-assisted Learning, Physics Education

I confirm that I have read this submission and believe that I have an appropriate level of expertise to confirm that it is of an acceptable scientific standard, however I have significant reservations, as outlined above.

# Response

**Published:** 2023-11-10

**Authors:** Gumisirizah Nicholus

**Affiliations:** African Centre of Excellence for Innovative Teaching and Learning Mathematics and Science (ACEITLMS), University of Rwanda, College of Education, (UR-CE), RWANDA, Rwanda

The article is scientifically well-structured. It demonstrates consistency in its research questions, methodology, results, discussion, and conclusions. The article also exhibits good originality, with no prior research similar to it. However, in the “Concept of PBL and its implementation” section, particularly concerning the implementation stages, ranging from problem identification to generalization, the inclusion of graphical illustrations would greatly enhance reader comprehension.

**Thank you so much! Graphical illustration for PBL implementation as in Figure 1.**

# Reviewer report: Reviewer response for version 1

**DOI:** 10.5256/f1000research.149476.r215455

**Published:** 2023-10-31

**Authors:** Elnetthra Folly Eldy

**Affiliations:** 1 Universiti Malaysia Sabah, Kota Kinabalu, Sabah, Malaysia

Dear Authors, 

 Overall the information presented represents valuable information regarding the role of PBL in teaching and learning physics particularly in students' academic achievement, attitude to learn physics, problem-solving, critical and creative thinking, cooperative learning enhancement, mental development, and science process skills attainment. The manuscript shows a lot of promise, but some minor details need to be addressed before it can be indexed.

1. I suggest you include the timeline of the articles you picked for this review. Does the article include within the past 10 years or more than that?
2. Does the filtering take into account the type of research method in each article? Does it include all types of research methods (i.e., quanti, quali, mixed methods) or only specific methods?
3. I suggest to resentence this, " *Other variables or skills not covered in the physics body of knowledge are reasoning abilities, metacognitive skills, lifelong learning skills, development of metacognition, self-efficacy, environmental literacy, higher-order thinking skills, management skills, and self-regulated learning. These are covered in other subjects apart from physics". *I believe, if you search carefully again, there are quite a number of journals that presented the impact of PBL on students' self-efficacy (for instance) in physics within past years. 
4. I suggest rechecking the grammar of the whole manuscript for more quality presentation. 

With the right changes, I believe that this manuscript can make a valuable contribution to the field of PBL in physics. I wish you good luck.

Are the rationale for, and objectives of, the Systematic Review clearly stated?

Yes

Is the statistical analysis and its interpretation appropriate?

Yes

Are sufficient details of the methods and analysis provided to allow replication by others?

Partly

Are the conclusions drawn adequately supported by the results presented in the review?

Yes

Reviewer Expertise:

Educational Technology in Physics

I confirm that I have read this submission and believe that I have an appropriate level of expertise to confirm that it is of an acceptable scientific standard, however I have significant reservations, as outlined above.

# Response

**Published:** 2023-11-10

**Authors:** Gumisirizah Nicholus

**Affiliations:** African Centre of Excellence for Innovative Teaching and Learning Mathematics and Science (ACEITLMS), University of Rwanda, College of Education, (UR-CE), RWANDA, Rwanda

Dear Authors,

 Overall the information presented represents valuable information regarding the role of PBL in teaching and learning physics particularly in students’ academic achievement, attitude to learn physics, problem-solving, critical and creative thinking, cooperative learning enhancement, mental development, and science process skills attainment. The manuscript shows a lot of promise, but some minor details need to be addressed before it can be indexed.

**Thank you so much for your encouragement! We have successfully followed your advice to frame our manuscript.**

 I suggest you include the timeline of the articles you picked for this review. Does the article include within the past 10 years or more than that?

**We added, “The study’s data analysis focused on articles spanning a diverse range of publication years, providing a comprehensive temporal perspective on the impact of PBL in physics education. Although a specific time frame was not set for the initial search, the analyzed data encompass articles published between 2004 and 2022. The selected studies encompassed various periods, with notable concentrations in 2010 and 2018, each contributing six and four articles, respectively. The distribution of articles across the years is as follows: 2004 (2 articles), 2009 (4 articles), 2011 (3 articles), 2012 (4 articles), 2014 (1 article), 2017 (2 articles), 2019 (3 articles), 2020 (3 articles), 2021 (1 article), and 2022 (3 articles). This temporal diversity allows for a nuanced examination of how the role and effectiveness of PBL in physics education have evolved over the past two decades.” In the method section, before the analysis paragraph.**

 Does the filtering take into account the type of research method in each article? Does it include all types of research methods (i.e., quanti, quali, mixed methods) or only specific methods?

**No, we included all types of research methods. We clarified that: “In the analysis of 36 selected articles, a predominant reliance on quantitative methods was observed, with 27 articles adopting this approach. A smaller subset of six articles opted for qualitative methods, highlighting a qualitative exploration of the problem space. Additionally, three articles utilized a mixed-methods approach, combining elements of both quantitative and qualitative methodologies.”  ** 

 I suggest to resentence this, “Other variables or skills not covered in the physics body of knowledge are reasoning abilities, metacognitive skills, lifelong learning skills, development of metacognition, self-efficacy, environmental literacy, higher-order thinking skills, management skills, and self-regulated learning. These are covered in other subjects apart from physics”. I believe, if you search carefully again, there are quite a number of journals that presented the impact of PBL on students’ self-efficacy (for instance) in physics within past years.

**We deleted “These are covered in other subjects apart from physics.” Sentence and inserted “widely” between “…skills not” and “covered in the physics body…” This adjustment acknowledges the possibility that some research has explored these variables within the context of physics education without making a definitive claim that they are entirely absent in the field.**

 I suggest rechecking the grammar of the whole manuscript for more quality presentation.

 We have checked grammatical errors throughout the whole manuscript.

 With the right changes, I believe that this manuscript can make a valuable contribution to the field of PBL in physics. I wish you good luck.

**Thank you so much once again!**

# Reviewer report: Reviewer response for version 1

**DOI:** 10.5256/f1000research.149476.r215446

**Published:** 2023-10-31

**Authors:** Sebatana M. Judicial

**Affiliations:** 1 Natural Sciences Education, North-West University, Gauteng, South Africa

This Systematic Review studies the role of Problem-Based Learning (PBL) as implemented during teaching and learning, with its main focus on the teaching and learning of Physics topics for the enhancement of 21st century skills.

 However, rationale for, and objectives of this Systematic Review are not clearly stated. The idea could be picked from the discussion but are not clearly stated. Therefore, they should be clearly stated.

 The second sentence under introduction section begins with "This approach..." which creates a gap since the approach in question have not been mentioned. Furthermore, it might help to connect PBL to the opening sentence. Again, the second line has "...problems are used...", it might be best to mention that the problems are real-life. The PBL process/characteristics must be omitted from introduction to avoid repetition. Citations by Karmila *et al., *2021 and Suwono and Wibowo 2018 emphasises the same idea, therefore must be restructured or combined.

 The choices of sites for articles in the methodology is not justified. Data analysis has not been fully described for simplicity when reading findings. This is also where the connection between objectives and results should be seen. The themes must be clearly stated and described.

 If is no trouble, the systematic review of PCK made by Vanessa Kind might be helpful in addressing most of these comments.

Are the rationale for, and objectives of, the Systematic Review clearly stated?

Partly

Is the statistical analysis and its interpretation appropriate?

Yes

Are sufficient details of the methods and analysis provided to allow replication by others?

Partly

Are the conclusions drawn adequately supported by the results presented in the review?

Yes

Reviewer Expertise:

Problem-Based Learning, PhET simulations and Blended Learning, 21st century skills, Pedagogical Content Knowledge, Open-Educational Resources and research methodologies.

I confirm that I have read this submission and believe that I have an appropriate level of expertise to confirm that it is of an acceptable scientific standard, however I have significant reservations, as outlined above.

# Response

**Published:** 2023-11-10

**Authors:** Gumisirizah Nicholus

**Affiliations:** African Centre of Excellence for Innovative Teaching and Learning Mathematics and Science (ACEITLMS), University of Rwanda, College of Education, (UR-CE), RWANDA, Rwanda

This Systematic Review studies the role of Problem-Based Learning (PBL) as implemented during teaching and learning, with its main focus on the teaching and learning of Physics topics for the enhancement of 21st century skills.

 However, rationale for, and objectives of this Systematic Review are not clearly stated. The idea could be picked from the discussion but are not clearly stated. Therefore, they should be clearly stated.

**We revised the last paragraph of the introduction, which incorporates our objective. “PBL is a dynamic educational method that challenges learners to engage with physics through real-world problem-solving, aligning theory with our daily experiences ( Ali, 2019). This approach empowers learners to become self-directed, fostering the acquisition of lifelong learning skills. PBL goes beyond the conventional learning, shaping critical thinkers and adapt problem solvers by seamlessly integrating physics knowledge and skills acquired during the teaching and learning process. Despite the prevalent perception of physics as a challenging subject among students ( Argaw et al., 2017), research indicates that PBL is a potent tool, significantly enhancing student achievement in physics.**

**The efficacy of PBL has been explored in various disciplines, such as medical studies ( Koh et al., 2008; Neville, 2008) and engineering education ( Boelt et al., 2022). Building on the foundation laid by Coppens et al. (2011), whose systematic review highlighted PBL’s effectiveness in promoting student learning outcomes, particularly in enhancing conceptual understanding. We recognize that the success of PBL hinges on factors like the quality of the presented problems, the level of student engagement, and the scaffolding and support provided by instructors.**

**Despite the strides made by researchers in implementing PBL, there remains a gap in understanding of specific variables learned under PBL and the skills it can accelerate in the teaching and learning processes. Consequently, our study is purposefully crafted to address a fundamental research question: What role does the PBL approach play in the context of teaching and learning physics? So, we aim to uncover the effectiveness of the PBL approach, using “role” and “effect” interchangeably throughout this manuscript.”**

 The second sentence under introduction section begins with “This approach...” which creates a gap since the approach in question have not been mentioned. Furthermore, it might help to connect PBL to the opening sentence. Again, the second line has “...problems are used...”, it might be best to mention that the problems are real-life. The PBL process/characteristics must be omitted from introduction to avoid repetition. Citations by Karmila et al., 2021 and Suwono and Wibowo 2018 emphasises the same idea, therefore must be restructured or combined.

**We started with “Problem-based learning (PBL) approach…” instead of “This approach …”**

**We added “real-life” in “problems are used…”**

**We deleted the “PBL is characterized by complex, real-world situations that do not have one correct answer ( Suwono and Wibowo, 2018)” to avoid repetition from what was presented by Karmila et al., 2021.**

 The choices of sites for articles in the methodology is not justified. Data analysis has not been fully described for simplicity when reading findings. This is also where the connection between objectives and results should be seen. The themes must be clearly stated and described.

**We explained the strengths of choosing our sites for articles: “Scopus and Web of Science are recognized for their inclusivity across various disciplines and their stringent peer-review processes, making them robust platforms for accessing high-quality academic literature.”**

**We revised the last paragraph of the introduction, which incorporates our objective, and revised the data analysis paragraph based on what we did in the results section. “Data analysis was conducted using Mendeley 1.19.5 / 2019 software, with subsequent organization and exploring variables through MS Excel 2016 to generate pivot tables and figures. The comprehensive analysis of each of the 36 studies aimed to identify the core concepts or content they focused on, along with the specific skills influenced or unaffected by PBL. We examined participant-level study designs and employed critical statistical methods as part of the process.**

**The analytical process involved a collective effort among all authors to ensure a unified focus. The primary author executed the data analysis, while each coauthor played a validating role at every step. The collective analysis primarily centered around deciphering the role of PBL in learning physics. Given the diverse manifestations of PBL’s impact, we applied a coding system to consolidate and simplify findings. For example, categories like student achievement, conceptual understanding, and performance were collectively coded as academic achievement.**

**To enhance readability and align with the study’s objectives, we systematically presented the conclusions regarding the impact of PBL on teaching and learning physics. The findings were categorized as either demonstrating a significant positive effect or indicating no substantial impact. In instances where various effects or roles emerged, we coded them clearly and consolidated them for better interpretation. For instance, attitudes toward learning physics were collectively coded to encompass motivation, appreciation, expectation, beliefs, perception, influence, and similar terms. Additionally, critical thinking, problem-solving, creative thinking, mental models, cooperative learning, and science process skills were coded individually to maintain precision and transparency in presenting themes.”**

 If is no trouble, the systematic review of PCK made by Vanessa Kind might be helpful in addressing most of these comments.

**Thank you. We have followed Vanessa Kind’s systematic review** **as guided**.

# Reviewer report: Reviewer response for version 1

**DOI:** 10.5256/f1000research.149476.r210612

**Published:** 2023-10-27

**Authors:** Jeanne Kriek

**Affiliations:** 1 Department of Physics, College of Science, Engineering and Technology, University of South Africa, Pretoria, Gauteng, South Africa

PBL is an interesting approach where students work in groups to solve an open-ended problem. In this article advantages of using PBL were offered, and the disadvantages were mentioned. However, it would be very interesting to find out in how many cases was it an open questions and students decided on the problem on their own without any support or in how many times were the problems suggested in one way or the other. From experience to develop a researchable question on a specific topic is not easy. Please add a column or analysis displaying this information – as this could inform the usage of PBL.

 Another suggestion is to provide the levels of the articles that you considered, for example, *how many* of the articles you considered were in high school and on tertiary level. You indicated that you considered all of them, but it would be interesting to show this in your analysis as focused recommendations could be made to different Departments of Basic Education in schools or Department of Higher Education. 

 In the paragraph on PBL and critical thinking there is a sentence that does not read well. “Science teachers’ experiences study...?.

Are the rationale for, and objectives of, the Systematic Review clearly stated?

Yes

Is the statistical analysis and its interpretation appropriate?

Yes

Are sufficient details of the methods and analysis provided to allow replication by others?

Yes

Are the conclusions drawn adequately supported by the results presented in the review?

Partly

Reviewer Expertise:

NA

I confirm that I have read this submission and believe that I have an appropriate level of expertise to confirm that it is of an acceptable scientific standard, however I have significant reservations, as outlined above.

# Response

**Published:** 2023-11-10

**Authors:** Gumisirizah Nicholus

**Affiliations:** African Centre of Excellence for Innovative Teaching and Learning Mathematics and Science (ACEITLMS), University of Rwanda, College of Education, (UR-CE), RWANDA, Rwanda

PBL is an interesting approach where students work in groups to solve an open-ended problem. In this article advantages of using PBL were offered, and the disadvantages were mentioned. However, it would be very interesting to find out in how many cases was it an open questions and students decided on the problem on their own without any support or in how many times were the problems suggested in one way or the other. From experience to develop a researchable question on a specific topic is not easy. Please add a column or analysis displaying this information – as this could inform the usage of PBL.

**Notably, the involvement of students in problem selection was limited, occurring in only one of the analyzed articles (Selçuk & Çalişkan, 2010). In this instance, students independently decided on the problem, showcasing a unique and student-driven approach to learning. Conversely, in ten articles, the suggestion of a problem was deemed not applicable, with a notable correlation to the prevalent use of qualitative methods in these cases. In most cases, the responsibility for deciding or suggesting a problem to students rested with the teacher. This was evident in diverse articles, where teachers took the lead in problem selection. This diversity in teacher-led problem introduction underlines educators’ choices in shaping their students’ learning experiences.**

**We provided the above information in the results section. We also provided implications and recommendations for this in the conclusion section: “Notably, student involvement in problem selection is limited, occurring in only one article, while teacher-centric decision-making prevails. Therefore, there are potential methodological biases and a need for greater student agency. Thus, it recommended promoting methodological diversity, fostering student-centered approaches, providing professional development for teachers, advocating for longitudinal studies, and encouraging collaboration within the physics education community.”.**

 Another suggestion is to provide the levels of the articles that you considered, for example, how many of the articles you considered were in high school and on tertiary level. You indicated that you considered all of them, but it would be interesting to show this in your analysis as focused recommendations could be made to different Departments of Basic Education in schools or Department of Higher Education.

**We already mentioned the number of articles for each educational level in the third sentence of the first paragraph under the results section; “Among 36 studies, 23 investigated university level; twelve were investigated in high schools and one in elementary school as in appendix A- (Analysis table of findings-**https://figshare.com/account/items/23573919/edit) **. Thirty-three studies used students, while three used teachers as participants.”**

**In the conclusion section, we then made a recommendation: “As revealed in our analysis** **across various educational levels, there is a predominant focus on the university setting, and there is an opportunity to expand research into the impact of PBL in high and elementary schools. Future studies should strive for a balanced representation across educational levels. Moreover, the limited focus on teachers as participants suggests a gap in understanding educators’ perspectives on implementing PBL. Further research in this area can offer valuable insights. Collaborative efforts between researchers and educators are encouraged to tailor PBL strategies to the unique needs of each educational level, promoting a more comprehensive and practical approach.”**

 In the paragraph on PBL and critical thinking there is a sentence that does not read well. “Science teachers’ experiences study...?.

**This was changed to: “The study on science teachers’ experience ….”**
